# Pancreatic stellate cells activated by mutant KRAS-mediated PAI-1 upregulation foster pancreatic cancer progression via IL-8

**DOI:** 10.7150/thno.36830

**Published:** 2019-09-23

**Authors:** Hao-Chen Wang, Yung-Lun Lin, Ching-Cheng Hsu, Ying-Jui Chao, Ya-Chin Hou, Tai-Jan Chiu, Po-Hsien Huang, Ming-Jer Tang, Li-Tzong Chen, Yan-Shen Shan

**Affiliations:** 1 Clinical Medicine Research Center, National Cheng Kung University Hospital, Tainan, Taiwan.; 2 Institute of Clinical Medicine, College of Medicine, National Cheng Kung University, Tainan, Taiwan.; 3 Department of Surgery, National Cheng Kung University Hospital, Tainan, Taiwan.; 4 Department of Medical Oncology, Kaohsiung Chang Gung Memorial Hospital, Kaohsiung, Taiwan.; 5 Graduate Institute of Clinical Medical Sciences, Chang Gung University College of Medicine, Kaohsiung, Taiwan.; 6 Department of Biochemistry and Molecular Biology, College of Medicine, National Cheng Kung University, Tainan, Taiwan.; 7 International Research Center of Wound Regeneration & Repair, National Cheng Kung University, Tainan, Taiwan.; 8 Department of Physiology, College of Medicine, National Cheng Kung University Tainan, Taiwan.; 9 National Institute of Cancer Research, National Healthy Research Institute, Tainan, Taiwan.

**Keywords:** pancreatic cancer, PSC, stiffness, organotypic coculture, PAI-1

## Abstract

**Background:** The dense fibrotic stroma enveloping pancreatic tumors is a major cause of drug resistance. Pancreatic stellate cells (PSCs) in the stroma can be activated to induce intra-tumor fibrosis and worsen patient survival; however, the molecular basics for the regulation of PSC activation remains unclear.

**Methods:** The *in vitro* coculture system was used to study cancer cell-PSC interactions. Atomic force microscopy was used to measure the stiffness of tumor tissues and coculture gels. Cytokine arrays, qPCR, and Western blotting were performed to identify the potential factors involved in PSC activation and to elucidate underlying pathways.

**Results:** PSC activation characterized by α-SMA expression was associated with increased pancreatic tumor stiffness and poor prognosis. Coculture with cancer cells induced PSC activation, which increased organotypic coculture gel stiffness and cancer cell invasion. Cancer cells-derived PAI-1 identified from coculture medium could activate PSCs, consistent with pancreatic cancer tissue microarray analysis showing a strong positive correlation between PAI-1 and α-SMA expression. Suppression by knocking down PAI-1 in cancer cells demonstrated the requirement of PAI-1 for coculture-induced PSC activation and gel stiffness. PAI-1 could be upregulated by KRAS in pancreatic cancer cells through ERK. In PSCs, inhibition of LRP-1, ERK, and c-JUN neutralized the effect of PAI-1, suggesting the contribution of LRP-1/ERK/c-JUN signaling. Furthermore, activated PSCs might exacerbate malignant behavior of cancer cells via IL-8 because suppression of IL-8 signaling reduced pancreatic tumor growth and fibrosis *in vivo*.

**Conclusions:** KRAS-mutant pancreatic cancer cells can activate PSCs through PAI-1/LRP-1 signaling to promote fibrosis and cancer progression.

## Introduction

Pancreatic cancer is an extremely aggressive disease with the mortality rate very close to the incidence rate. Globally, there were 458,918 cases of newly diagnosed pancreatic cancer in 2018 with 432,242 people dying from this disease 1. Drug resistance is one of the major contributing factors for the miserable outcome in pancreatic cancer patients and is partially due to the lack of efficient chemotherapy drug delivery to neoplastic cells 2. Pancreatic cancer is characterized by the abundant desmoplastic stroma around tumors, making up over 90% of the tumor mass. The desmoplastic reaction has been suggested to be caused by the interactions between cancer cells and their adjacent stroma cells, which promotes the proliferation of myofibroblast-like cells to increase the production of extracellular matrix (ECM) components and thus leads to increased fibrosis in cancer tissues 3. The dense fibrosis creates physical barriers against the delivery of conventional drugs. Therefore, strategies for overcoming drug resistance in pancreatic cancer may include breakdown of desmoplasia to improve drug delivery.

The pancreatic tumor stroma comprises ECM and diverse types of noncancerous cells including fibroblasts, stellate cells, endothelial cells, and immune cells 2. Pancreatic stellate cell (PSC), a predominant cellular component of pancreatic cancer stroma, is a key driver in the desmoplastic reaction of chronic pancreatitis and pancreatic cancer 4-6. In normal pancreas, PSCs reside in a quiescent state, which are characterized by the accumulation of vitamin A containing lipid droplets in the cytoplasm. In response to pancreatic injury or inflammation, quiescent PSCs undergo a transition into a myofibroblast-like phenotype featured by the expression of cytoskeletal protein α-smooth muscle actin (α-SMA). This conversion process is called PSC activation. Activated PSCs have been identified as the predominant source of the ECM proteins that are excessively deposited during pancreatic fibrogenesis 7. Evidence accumulating from in vitro coculture experiments has shown that pancreatic cancer cells have the potential to activate PSCs, which in turn reciprocate by enhancing proliferation, migration, and survival of pancreatic cancer cells 4, 8. Animal studies using the orthotopic pancreatic cancer xenograft mouse model have demonstrated that the presence of PSCs facilitates tumor growth and metastasis 8. Intriguingly, PSCs can be also found to accompany pancreatic cancer cells to metastatic sites and stimulate angiogenesis, suggesting that PSCs can seed in distant organs to form a metastatic niche. Collectively, these results convincingly demonstrate the protumoral role for activated PSCs; however, the molecular mechanism responsible for the activation of PSCs remains to be investigated.

ECM is a major and essential component of the tumor microenvironment (TME). High ECM rigidity resulting from excessive ECM deposition and increased crosslinking was frequently observed in cancer tissue 9. Recent research has demonstrated that the stiffness of tumor ECM plays a critical role in promoting cancer progression. The proliferation of cancer cells interacting with stiffer ECM in response to growth stimuli is stronger, and FAK, ERK, PI3K, and RAC can be activated to upregulate cyclin D1 10-12. Besides triggering oncogenic signaling, ECM stiffness can also inhibit the tumor suppressor genes PTEN and GSK3 through microRNAs to promote malignancy. These findings make tumor ECM stiffness an appealing therapeutic target.

Plasminogen activator inhibitor-1 (PAI-1), also known as SERPINE1, is a member of the serine protease inhibitor (SERPIN) protein family and plays a vital role in regulating blood coagulation, cell migration, cell invasion, and apoptosis 13, 14. To exert its biological activities, PAI-1 binds to its cell surface receptors identified over the years, including low-density lipoprotein receptor-related protein-1 (LRP-1), tissue-type plasminogen activator receptor (tPAR), and urokinase-type plasminogen activator receptor (uPAR) 15, 16. As a serine protease inhibitor, PAI-1 can suppress fibrinolysis by inactivating tPA and uPA 17. In addition, PAI-1 also promotes fibrogenesis in multiple organs, including lung and kidney, demonstrating the profibrotic role for PAI-1 18, 19. In the past years, PAI-l has been linked to cancer because several studies revealed a paradoxical association of increased PAI-1 expression in cancer with unfavorable clinical outcomes and poor response to therapy 20, 21. A previous animal study has found high expression of PAI-1 in the primary pancreatic tumor, the tumor invasion front, and the metastases 22. Depletion of PAI-1 in pancreatic cancer cells increased E-cadherin expression and promoted epithelial phenotypic changes, supporting its negative impact on cancer patient outcome 23.

In this study, we have found that coculture with pancreatic cancer cells increased PSC activation. The cytokine array identified a significant upregulation of PAI-1 in the conditioned medium (CM) of pancreatic cancer cell/PSC coculture. By using patient tissue materials and *in vitro* coculture experiments, we aimed to determine the function of PAI-1 in PSC activation and pancreatic cancer stiffness and to explore the underlying mechanism.

## Materials and Methods

### Cell culture

The human pancreatic cancer cell lines PANC-1, Mia PaCa-2, AsPC-1, and BxPC-3, were obtained from the American Type Culture Collection. The human PSC cell line RLT-PSC immortalized by SV40 large T antigen was given by Dr. Kelvin K. Tsai (National Institute of Cancer Research, National Health Research Institutes, Taiwan). Cells were maintained in DMEM medium with 10% fetal bovine serum (FBS; Hyclone^TM^) and 1% antibiotic-antimyocotic solution (*Caisson* Laboratories) and incubated at 37°C in a humidified atmosphere containing 5% CO2. RLT-PSCs were maintained in an inactivation status using N-acetylcysteine (NAC) prior to coculture with cancer cells or PAI-1 treatment.

### Transgenic mice

*Pdx1-Cre* mice and *LSL-Kras^G12D^* mice were purchased from the Jackson Laboratory. *Trp53^fl/fl^* mice were provided by Prof. Kuang-Hung Cheng (Institute of Biomedical Sciences, National Sun Yat-Sen University, Kaohsiung, Taiwan). The *Trp53^fl/fl^* mice were crossed with the *Pdx1-Cre* mice to generate *Trp53^fl/fl^*;*Pdx-Cre* offspring, and the *LSL-Kras^G12D^* mice were crossed with *Trp53^fl/fl^* mice to generate *LSL-Kras^G12D^*;*Trp53^fl/fl^* offspring. Finally, the *Trp53^fl/fl^*;*Pdx-Cre* mice were crossed with the *LSL-Kras^G12D^*;*Trp53^fl/fl^* mice to generate the *LSL-Kras^G12D/+^*;*Trp53^fl/fl^*;*Pdx1-Cre* (termed KPC) mice that were genotyped by PCR and screened for the presence of pancreatic tumors by ultrasound at 4 weeks of age. The KPC mice were randomly divided into the control group (10% DMSO in 1x PBS, intraperitoneal (IP) injection) and the SB225002 group (0.3 mg/kg, IP injection, 3 times per week). All mice were housed under pathogen free conditions and had free access to water and food. All experimental protocols were approved by the Institutional Animal Care and Use Committee of the National Cheng Kung University.

### Transfection

To generate mutant KRAS overexpressing cells, the pcDNA3.1 *KRAS^G12D^* plasmid, a gift from Prof. Ming-Derg Lai (Department of Biochemistry and Molecular biology, College of Medicine, National Cheng Kung University, Tainan, Taiwan), was transfected into BxPC-3 cells using HyFect^TM^ DNA transfection reagent (Leadgene Biomedical) according to the manufacturer's protocol. Forty-eight hours after transfection, G418 (200 μg/mL, Sigma-Aldrich) was used for selection and maintenance thereafter. The transfection efficiency was determined by Western blotting.

For transient transfection of siRNA, *JUN* siRNA (accession number NM_002228.3; Invitrogen) was transfected into RLT-PSCs using HyFect^TM^ DNA transfection reagent (Leadgene Biomedical). Forty eight hours after transfection, the knockdown efficiency was monitored by Western blotting.

### Viral infection

To knock down KRAS and PAI-1 in pancreatic cancer cells and LRP-1 in PSCs, cells were infected with sh*KRAS*, sh*PAI-1*, sh*LRP-1*, and sh*LUC* (control) lentiviral particles (National RNAi Core Facility, Academia Sinica, Taipei, Taiwan) in the presence of polybrene (5 μg/mL; Sigma-Aldrich) for 24 hours. Puromycin (Sigma-Aldrich) was used for drug selection of infected cells to generate permanent cell lines. The knockdown efficiency was checked by Western blotting.

### Patients and tissue microarray (TMA)

The collection of pancreatic cancer specimens was approved by the Institutional Review Board of National Cheng Kung University Hospital (NCKUH). Patients were prospectively followed up until death or 2012. Anonymous archived pancreatic cancer samples from 91 patients, including both normal and tumor tissues, were obtained from Human Biobank of NCKUH for TMA construction.

### Immunohistochemistry (IHC)

Formalin-fixed and paraffin-embedded human and mouse pancreatic tumor tissue blocks were cut into 4μM-thick sections and applied to silanized slides. The slides were incubated in blocking solution for 30 minutes and then stained with the primary antibody against α-SMA (Genetex) at 4°C overnight. Next day, the slides were incubated with the secondary antibody at 25°C for 30 minutes. The stain in the slides was developed by incubation with DAB, and the sections were counterstained with hematoxylin. Images were obtained using a BX51 microscope (OLYMPUS).

### Masson's trichrome staining

Serial sections of human and mouse pancreatic tumor tissues were stained for collagen with the Masson's trichrome stain kit (Sigma-Aldrich) according to the manufacturer's protocol. Hematoxylin was used to counterstain the cell nuclei. Bright-field images were captured using a BX51 microscope (OLYMPUS).

### Simplified coculture system

A transwell insert with 0.4 μm pore size (BD Bioscience) was used for the coculture experiments. Cancer cells (1×10^5^/well) were seeded in the upper chamber inserts and RLT-PSCs (1×10^5^/well) were seeded in the lower chambers. The cocultures were incubated for 72 hours before further assays.

### Organotypic coculture system

The organotypic culture system was developed as described previously with minor modifications 24. Briefly, the RLT-PSC cell suspensions (5x10^5^/mL) were mixed with 3 mL gel (mixture of 10x EMEM, 200 mM L-glutamine, FBS, 2.5% sodium bicarbonate, and collagen I) and then placed into 6-well inserts. After 5 days of gelling, cancer cells (5x10^5^/mL) were seeded on the top of the gel. Cancer cells cultured on the surface of the gel without RLT-PSCs were regarded as a control. The gels were harvested after 10 days of coculture to measure the stiffness.

### Cytokine array

Proteins secreted in coculture medium were identified using the Human Cytokine Array Kit (Ary005, R&D Systems) according to the manufacturer's instructions. Briefly, the CM of RLT-PSC monoculture and PANC-1 cell/RLT-PSC coculture were collected, filtered, and mixed with a cocktail of biotinylated detection antibodies. The mixtures were then incubated with the array membrane spotted in duplicate with capture antibodies to specific target proteins. The protein spots were detected using Western Lighting Plus-ECL (Perkin Elmer), digitally captured using the Biospectrum Imaging System (UVP), and quantified using the Gel-Pro Analyzer software (Media Cybernetics).

### RNA preparation and quantitative real-time polymerase chain reaction (qPCR)

Total RNA was isolated using the Total RNA Miniprep Purification Kit (Genemark Technology) according to the manufacturer's protocol. One μg of RNA was reverse transcribed for each cDNA using 1.0 uM oligo-dT and Deoxy^+^ HiSpec reverse transcriptase (Yeasterm Biotech). A 1/20 volume of the cDNA mixture was subjected to qPCR using the GoTaq real-time PCR systems (Promega). The sequences of qPCR primers were as follows: PAI-1 sense primer, 5'-CACAAATCAGACGGCAGCAC-3', PAI-1 antisense primer, 5'-GAGCTGGGCACTCAGAATGT-3', collagen I sense primer, 5'-AAAGAAGGCGGCAAAGGTC-3', collagen I antisense primer, 5'-GTCCAGCAATACCTTGAGGC-3', fibronectin sense primer, 5'-ATTTGCTCCTGCACATGCTT-3', fibronectin antisense primer, ACTCTCGGGAATCTTCTCTGT-3', GAPDH sense primer, 5'-GGGGCTCTCCAGAACATCAT-3', and GAPDH antisense primer, 5'-GTCGTTGAGGGCAATGCCAG-3'. The reaction protocol was set as follows: an initial denaturation at 95°C for 5 minutes followed by 40 cycles of denaturation at 95°C for 10 seconds, annealing at 60°C for 10 seconds, and extension at 72°C for 10 seconds. During the annealing-extension step, the StepOne^TM^ monitored real-time PCR amplification by quantitatively analyzing fluorescence emissions. GAPDH was used as an internal control.

### Western blot analysis

Equal amounts of total protein were separated by sodium dodecyl sulfate-polyacrylamide gel electrophoresis (SDS-PAGE) and transferred to polyvinylidene difluoride membranes (Millipore). The membranes were blocked in 5% non-fat milk at room temperature for 1 hour followed by incubation with primary antibodies at 4°C overnight. Next day, the membranes were incubated with HRP-conjugated secondary antibodies at room temperature for 1 hour. The blot was developed using Western Lighting Plus-ECL (Perkin Elmer) and visualized using the Biospectrum Imaging System (UVP).

### Measurement of cell growth

Cell growth was analyzed by the 3-(4,5-dimethylthiazol-2-yl)-2,5-diphenyl-tetrazolium bromide (MTT) assay. Briefly, cells were seeded in 96-well plates (3×10^3^ cells/well). After treatments, MTT (Sigma-Aldrich) stock solution (5 mg/mL in PBS) was added to (final concentration of 0.5 mg/mL) each well, and plates were incubated for 4 hours at 37°C to allow MTT to form purple formazan crystals in metabolically active cells. After centrifugation, MTT solution and medium were aspirated from the wells and DMSO was added to dissolve the crystals. The absorbance of the solutions was measured at a wavelength of 490 nm with an ELISA reader (Dynatech Laboratories).

### Migration assay

Migration assays were conducted using a 24-well transwell chamber (Corning Coster). PSCs were seeded (2x10^4^ cells in 0.1 mL DMEM medium) in the upper chamber, and medium containing PAI-1 (200 ng/mL; R&D Systems) or the CM derived from cancer cells was placed in the bottom well. After incubation at 37°C for 8 hours, cells on the lower surface were fixed with 4% paraformaldehyde and then stained with crystal violet (Sigma-Aldrich). Migrating cells that have reached the underside of the membrane were counted in 10 randomly chosen fields of view under a light microscope to get an average number.

### Immunofluorescence (IF) staining

The human pancreatic cancer TMA slides were incubated with blocking solution for 30 minutes. Next, the slides were stained with primary antibodies against α-SMA and PAI-1 (Genetex) at 4°C overnight followed by incubation with secondary antibodies at 25°C for 30 minutes. Cell nuclei were counterstained with DAPI. The images of TMAs were obtained from the FACS-like Tissue Cytometry. The percentages of positive cells in each sample were further quantified by TissueQuest software (TissueGnostics) as described previously 25. For IF staining in RLT-PSCs, cells were fixed using 4% paraformaldehyde followed by incubation with oil red O for 15 minutes after PAI-1 treatment (200 ng/mL; R&D Systems) or coculture with PANC-1 cells. RLT-PSCs were washed with ddH_2_O and incubated with blocking solution for 30 minutes. Next, RLT-PSCs were stained with primary antibodies against α-SMA at 4°C overnight and then incubated with fluorescent-dye conjugated secondary antibodies at 25 °C for 30 minutes. Nuclei were counterstained with DAPI. All the images were visualized using a fluorescence microscope (BX51, OLYMPUS).

### Atomic force microscopy (AFM)

For measurements of the stiffness of cells or tissues, the NanoWizard^®^ II AFM with BioCell (JPK Instrument) was equipped and manipulated as previously described 26. Tumor tissues were embedded in OCT compound and then sliced into sections with 30 µm in thickness. The sections were subsequently put onto glass slides and immersed in PBS supplemented with protease inhibitor. The 20 μm (in diameter) polystyrene bead-modified tip-less cantilever (CSC12, MikroMasch) with nominal spring constant 0.3N/m was used to generate 3nN applied force with 1 µm/second approaching/retracting speed (to avoid the effects of hydrodynamics on AFM assessment) on pancreatic tissues. After cells were cultured in collagen gel on 6-well inserts, the stiffness of the collagen gel was measured by AFM. The 5 μm-diameter polystyrene bead-modified tip-less cantilever (ARROW-TL1-50, NanoWorld) with nominal spring constant 0.03N/m was used to apply 1nN with 1 µm/second approaching/retracting speed on collagen gels. All the force-displacement curves obtained from pancreatic tissues and collagen gels were analyzed using the JPK package software, from which the effective Young's modulus of the pancreatic tissues and collagen gels were calculated based on the Hertz model 27. At least 60 points of indentation results were collected in each experiment.

### Statistics

Numerical data were represented as the mean ± SEM of triplicate determinations. Comparisons were analyzed using GraphPad Prism 5.0 (GraphPad software) and SPSS Statistics 17.0 (IBM software). *P* value < 0.05 was regarded as statistically significant. Overall survival (OS) and disease-free survival (DFS) were analyzed with the Kaplan-Meier method and the differences were calculated using log-rank test.

## Results

### PSC activation increases pancreatic cancer tissue stiffness and predicts poor survival

**Figure 1:**
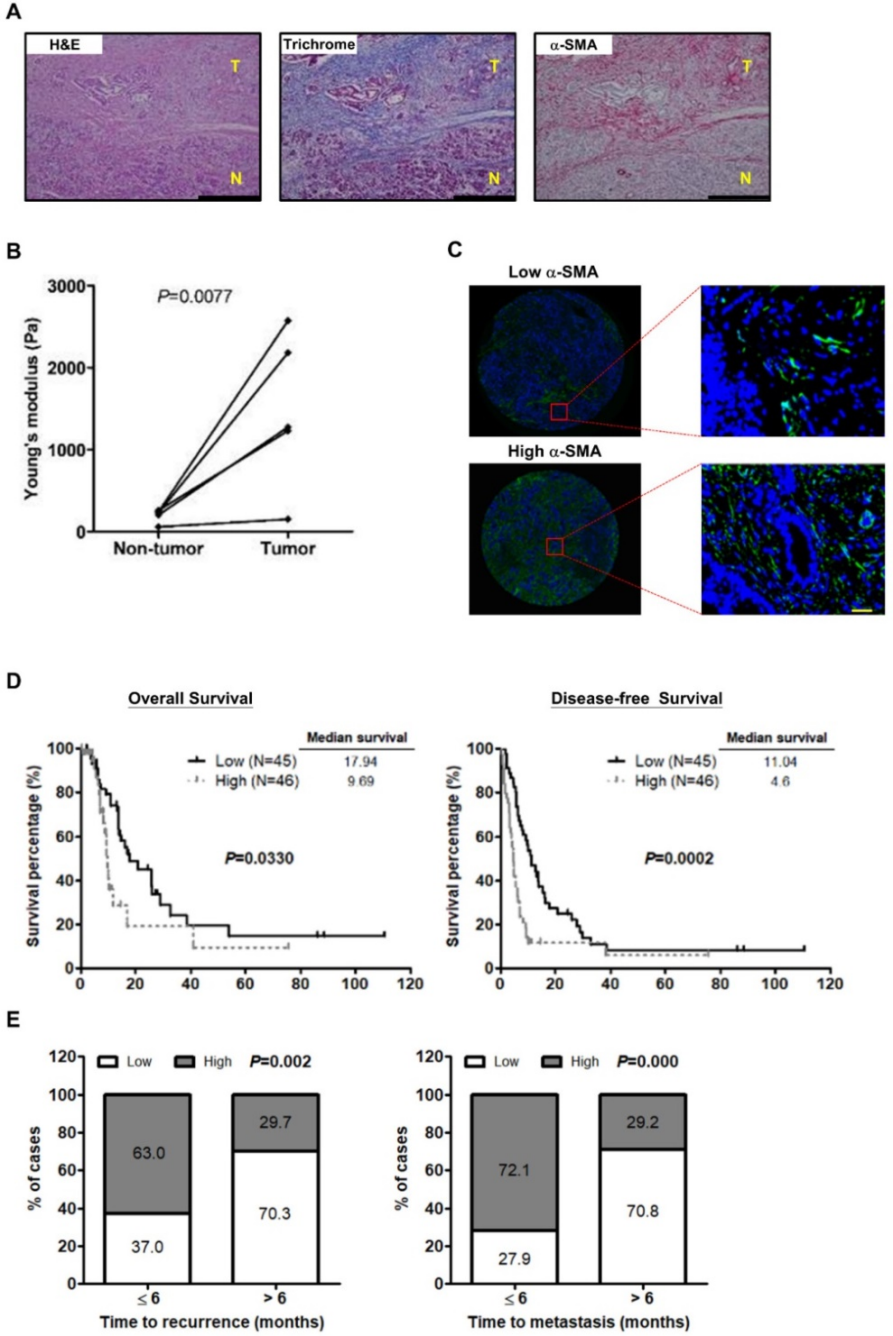
** PSC activation is associated with the stiffness of pancreatic cancer tissue and poor outcome in patients. A,** Expression of α-SMA and collagen in human pancreatic cancer tissue was determined by IHC and Masson's trichrome stain. Magnification: 200x, scare bar: 200 μm. **B,** The stiffness of non-tumor tissues and tumor tissues from the paired specimens of 5 patients was measured by AFM and compared. *P*=0.0077, paired t-test. **C,** Expression of α-SMA was analyzed by IF staining in TMAs of 91 pancreatic tumors. Patients were divided into the low expression group (left) and the high expression group (right) according to the mean percentage of α-SMA positive cells as cutoff point at 55.8%. Images on the bottom panel are high-magnification (200x; scale bar: 20 μm) of areas outlined by red squares. **D,** The correlations of α-SMA expression with patient OS and DFS were analyzed using Kaplan-Meier survival analysis. **E,** The bar graph depicts the frequency distribution of low and high α-SMA expression for early recurrence and late recurrence (left; *P*=0.002, Chi-square Test) and for early metastasis and late metastasis (right; *P*=0.000, Chi-square Test).

Recent studies have shown that activated PSCs induce ECM synthesis to drive fibrosis in pancreatic cancer 28. We preformed Masson's trichrome staining for collagen and IHC staining for the activated PSC marker α-SMA on paired pancreatic tumor/normal tissue samples to confirm the effect of PSC activation on ECM deposition. Increased PSC activation and collagen expression were observed in the tumor tissues in comparison with the non-tumor counterparts. Additionally, both α-SMA and collagen were localized in the same areas of the specimens (Figure 1A), demonstrating activated PSCs as a predominant source of ECM. We further investigated whether PSC activation increases the stiffness of pancreatic cancer tissues. The results show that tumor tissues were stiffer than the corresponding non-tumor parts (Figure 1B), revealing a positive correlation between PSC activation and tumor stiffness.

Next, to assess the effect of PSC activation on patient survival, IF staining for α-SMA on human pancreatic cancer TMAs was conducted. The clinicopathologic characteristics of pancreatic cancer patients collected for TMA construction were shown in Table S1. The mean percentage of α-SMA-expressing cells, 55.5% was used as a cutoff point for the classification of patients into two groups: the high α-SMA expression group (≥ mean value) and the low α-SMA expression group (< mean value) (Figure 1C). The patients in the high α-SMA expression group had worse OS and DFS (Figure 1D). High α-SMA expression was associated with a greater incidence of early disease recurrence and early metastasis after dichotomization of recurrence time or metastasis time using 6 months post-surgery as a cut off (Figure 1E).

### PAI-1 secreted from pancreatic cancer cells induces PSC activation

**Figure 2:**
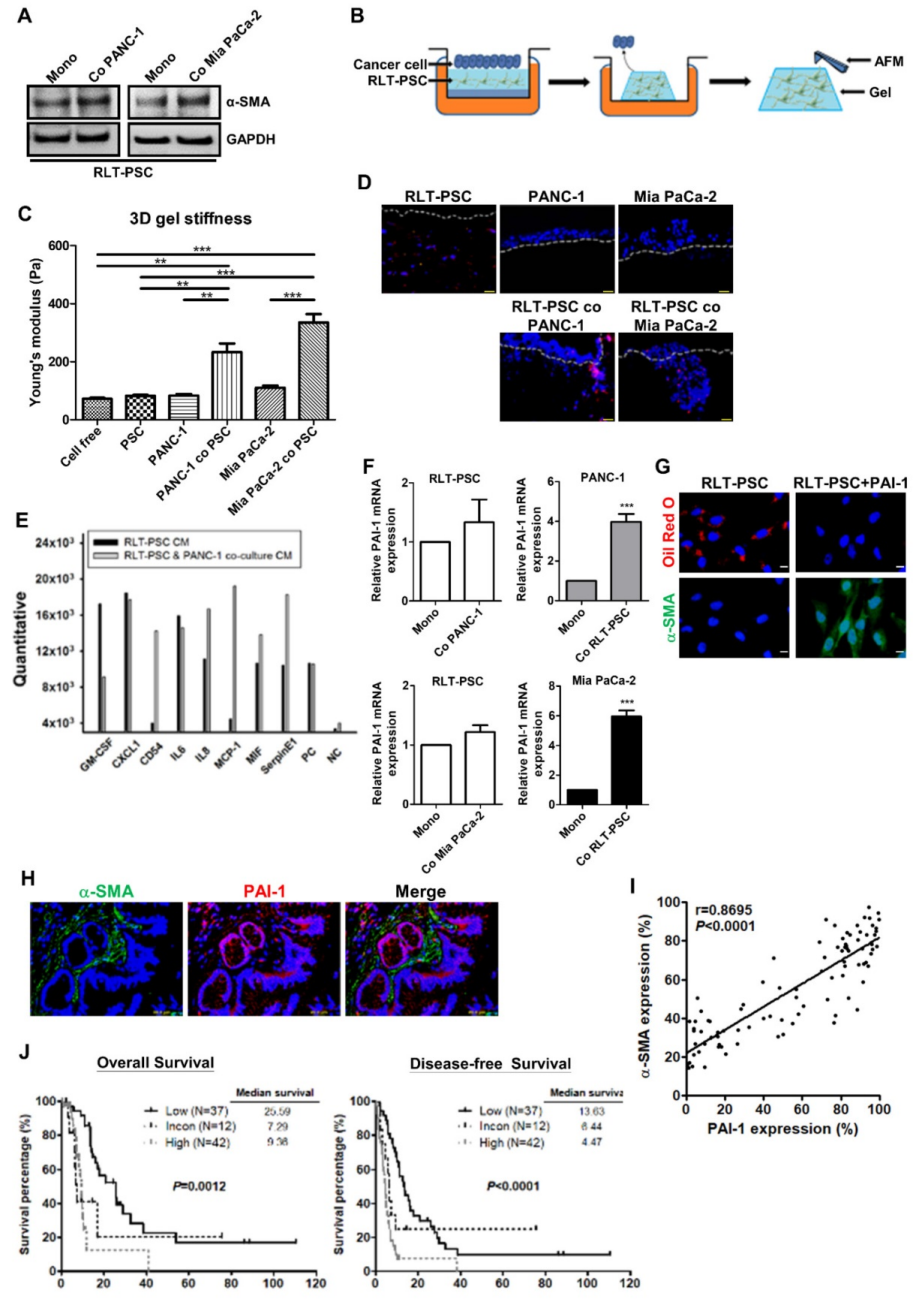
** PAI-1 is involved in pancreatic cancer cells-induced PSC activation and confers poor patient outcome. A,** After monoculture or coculture with PANC-1 or Mia PaCa-2 cells in a simplified coculture system for 72 hours, α-SMA expression in RLT-PSC cells were determined by Western blotting. **B,** The schematic representation of a 3D organotypic coculture model. AFM was used to measure the stiffness of organotypic gels. **C,** The stiffness of organotypic gels of monocultures or cocultures was measured by AFM. The bar graph indicates the average values of Young's modulus of organotypic gels. ** *P* < 0.01; *** *P* < 0.001, significant difference between groups, one-way ANOVA. **D,** PANC-1 and Mia PaCa-2 cells were monocultured or cocultured with RLT-PSC cells carrying red fluorescent protein in 3D gels. Cell *nuclei were* stained with *DAPI.* The gels were visualized using fluorescence microscopy. The dotted white lines indicate the upper surface of the organotypic gels. **E,** The CM of RLT-PSC monoculture and RLT-PSC/PANC-1 coculture were collected for cytokine arrays. The bar graph shows differential expression of cytokines. **F,** After coculture, mRNA in PANC-1, Mia PaCa-2, and RLT-PSC cells was collected to measure PAI-1 gene expression using qPCR. The bar graphs depict the relative expression of PAI-1 mRNA. *** P < 0.001 versus monoculture control, unpaired t-test. **G,** After treatment with PAI-1 (200 ng/mL) for 24 hours, RLT-PSC cells were stained with anti-α-SMA antibodies (green) and oil red O (red). Magnification: 400x, scale bar: 20 μm. **H,** The human pancreatic tumor TMA sections were stained with anti-PAI-1 (red) and anti-α-SMA (green) antibodies. Magnification: 200x, scale bar: 20 μm. **I,** The correlation between PAI-1 and α-SMA expression was analyzed by Pearson correlation coefficient test (r=0.8695; *P* < 0.0001). **J,** The association between patient survival (OS and DFS) and the combination of PAI-1 and α-SMA expression was determined by Kaplan-Meier survival analysis.

Accumulating evidence has suggested that pancreatic cancer cells can stimulate PSC activation 4. Here, we show that coculture with PANC-1 or Mia PaCa-2 cells in a simplified coculture system enabled RLT-PSCs to undergo activation featured by increased α-SMA expression (Figure 2A). To investigate the effect of PSC activation on tissue stiffness and cancer aggressiveness, the organotypic 3D coculture system of pancreatic cancer cells and RLT-PSC cells was adopted. Figure 2B shows the schematic representation of an organotypic coculture model described previously 24. The organotypic gel stiffness of pancreatic cancer cell/RLT-PSC cocultures measured by AFM was higher than that of RLT-PSC monoculture or cancer cell monocultures (Figure 2C). After monoculture, PANC-1 cells and Mia PaCa-2 cells remained on the surface of 3D gels; however, coculture with RLT-PSCs increased cancer cell invasion into the gel (Figure 2D). These data suggested that pancreatic cancer cells can activate PSCs, which increase matrix stiffness and in turn exacerbate the malignant behavior of cancer cells.

To determine the factors contributing to PSC activation, the CM from coculture of RLT-PSCs and PANC-1 cells was collected for cytokine array. As compared with the CM of RLT-PSC monoculture, several cytokines, such as CD54, IL8, MCP-1, MIF, and PAI-1, were upregulated in the coculture medium (Figure 2E). Because PAI-1 has been implicated in the pathology of fibrosis, we focused on its role in PSC activation. To identify the cellular source of the elevated level of PAI-1, PAI-1 mRNA expression in PANC-1, Mia PaCa-2, and RLT-PSC cells before and after coculture was analyzed. After coculture, PAI-1 mRNA expression was increased in PANC-1 and Mia PaCa-2 cells but not altered in RLT-PSCs (Figure 2F), suggesting that pancreatic cancer cells are the major source of PAI-1 production. We further verified whether PSCs can be activated by PAI-1. Treatment with PAI-1 recombinant protein in RLT-PSCs increased α-SMA expression and resulted in loss of *vitamin* A-containing lipid droplets (Figure 2G), suggesting the involvement of PAI-1 in PSC activation.

### High expression of PAI-1 and α-SMA is associated with poor patient outcomes

To address the clinical significance of PAI-1 in pancreatic cancer, the correlation between PSC activation and PAI-1 expression was analyzed and its influence on patient survival was evaluated. Double IF staining for α-SMA (green) and PAI-1 (red) was performed on pancreatic cancer TMAs. Activated PSCs characterized by high α-SMA expression surrounded PAI-1^+^ tumor cells (Figure 2H). There was a strong positive correlation between α-SMA and PAI-1 expression (Figure 2I). To assess the prognostic value of the combination of PAI-1 and α-SMA expression in pancreatic cancer, we divided the patients into 3 groups: the high expression group (staining percentages of PAI-1 ≥ the mean value of 56% and α-SMA ≥ the mean value of 55.5%; N=42), the low expression group (staining percentages of PAI-1 < 56% and α-SMA < 55.5%; N=37) , and the inconsistent group (staining percentages of PAI-1 ≥ 56% and α-SMA < 55.5% or staining percentages of PAI-1 < 56% and α-SMA ≥ 55.5%; N=12). As compared with the low expression group and the inconsistent group, both OS and DFS were significantly worst in the high expression group (Figure 2J).

### PAI-1 knockdown attenuates coculture-induced PSC activation and organotypic gel stiffness

**Figure 3:**
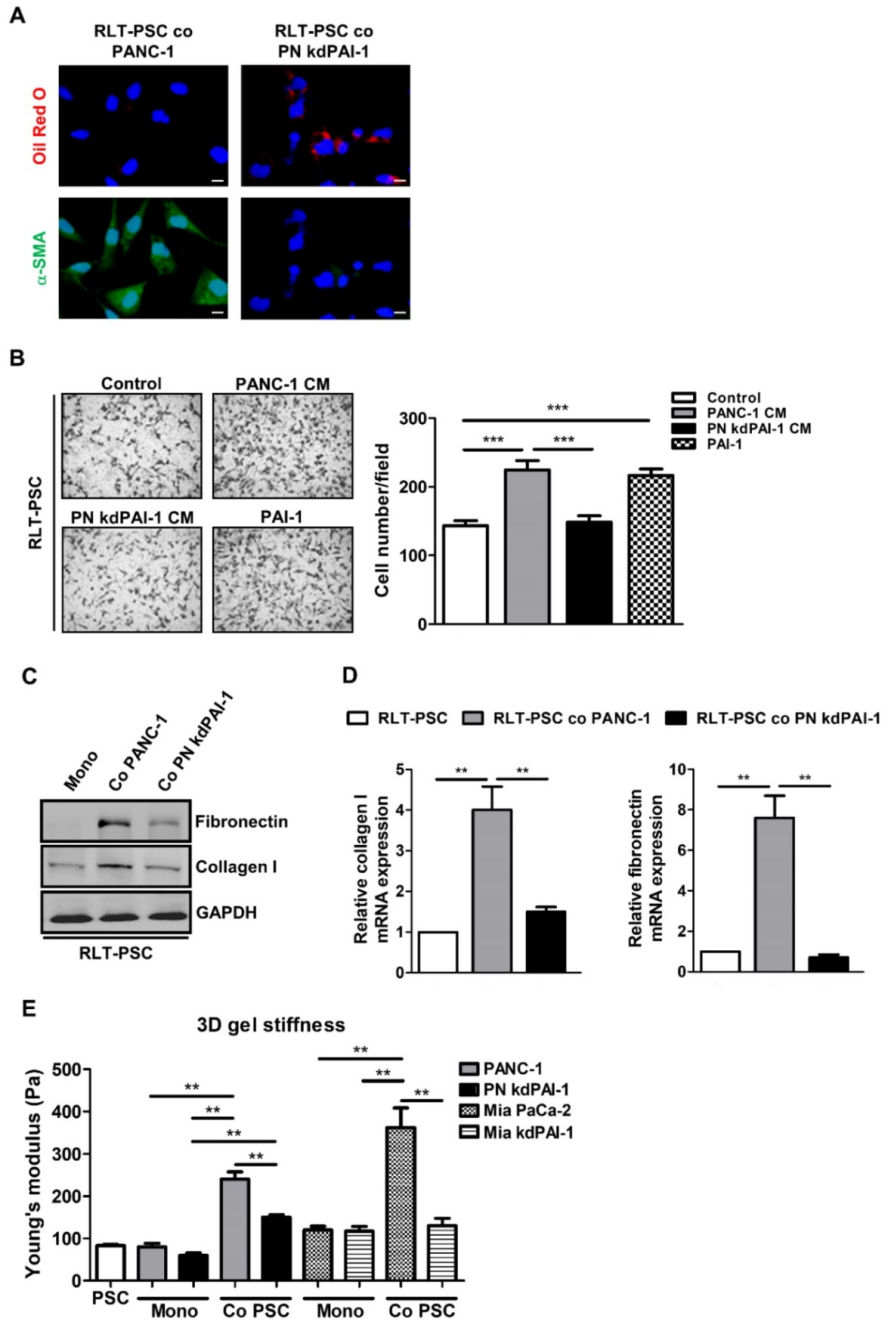
** Knockdown of PAI-1 counteracts coculture-induced PSC activation and gel stiffness. A,** After coculture with PANC-1 or PN kdPAI-1 cells for 72 hours, RLT-PSC cells were stained with anti-α-SMA antibodies (green) and oil red O (red). Magnification: 400x, scale bar: 20 μm. **B,** The migration of RLT-PSC cells was analyzed using transwell assays after 24 hours of incubation with the CM from PANC-1 and PN kdPAI-1 cells or fresh medium containing PAI-1 (200 ng/mL). The bar graph indicates the average number of migrating cells. *** *P* < 0.001, significant difference between groups, one-way ANOVA. **C and D,** Protein and mRNA expression of collagen I and fibronectin in RLT-PSC cells after coculture with PANC-1 or PN kdPAI-1 cells was determined by Western blotting and q-PCR. The bar graphs depict the relative expression of collagen I and fibronectin mRNA. ** *P* < 0.01, significant difference between groups, one-way ANOVA. **E,** After monoculture or coculture, the stiffness of 3D gels was measured by AFM. The bar graph indicates the average values of Young's modulus of organotypic gels. ** *P* < 0.01, significant difference between groups, two-way ANOVA.

To determine the importance of PAI-1 in PSC activation, a stable knockdown cell line PN kdPAI-1 was established from PANC-1 cells using PAI-1 shRNA. Coculture with PANC-1 cells increased α-SMA expression and reduced accumulation of vitamin A-containing lipid droplets in RLT-PSCs, but coculture with PN kdPAI-1 cells reversed this effect (Figure 3A). After activation, PSCs can acquire strong abilities to migrate and synthesize a large amount of ECM proteins, such as fibronectin and collagen 29, 30, so we examined the effect of PAI-1 on cell migration and ECM production in RLT-PSCs. Treatment with exogenous PAI-1 or the CM of PANC-1 cells enhanced the migration of RLT-PSCs, but treatment with the CM of PN kdPAI-1 cells did not (Figure 3B). Expression of collagen I and fibronectin was induced in RLT-PSCs after coculture with PANC-1 cells, whereas the induction was suppressed by knockdown of PAI-1 (Figure 3C and 3D). In addition, silencing PAI-1 reduced coculture-mediated increase in stiffness of the organotypic gels (Figure 3E). These results demonstrate the requirement of PAI-1 for coculture-induced PSC activation and matrix stiffness.

### Upregulation of PAI-1 is induced by *KRAS* mutation in pancreatic cancer cells

**Figure 4:**
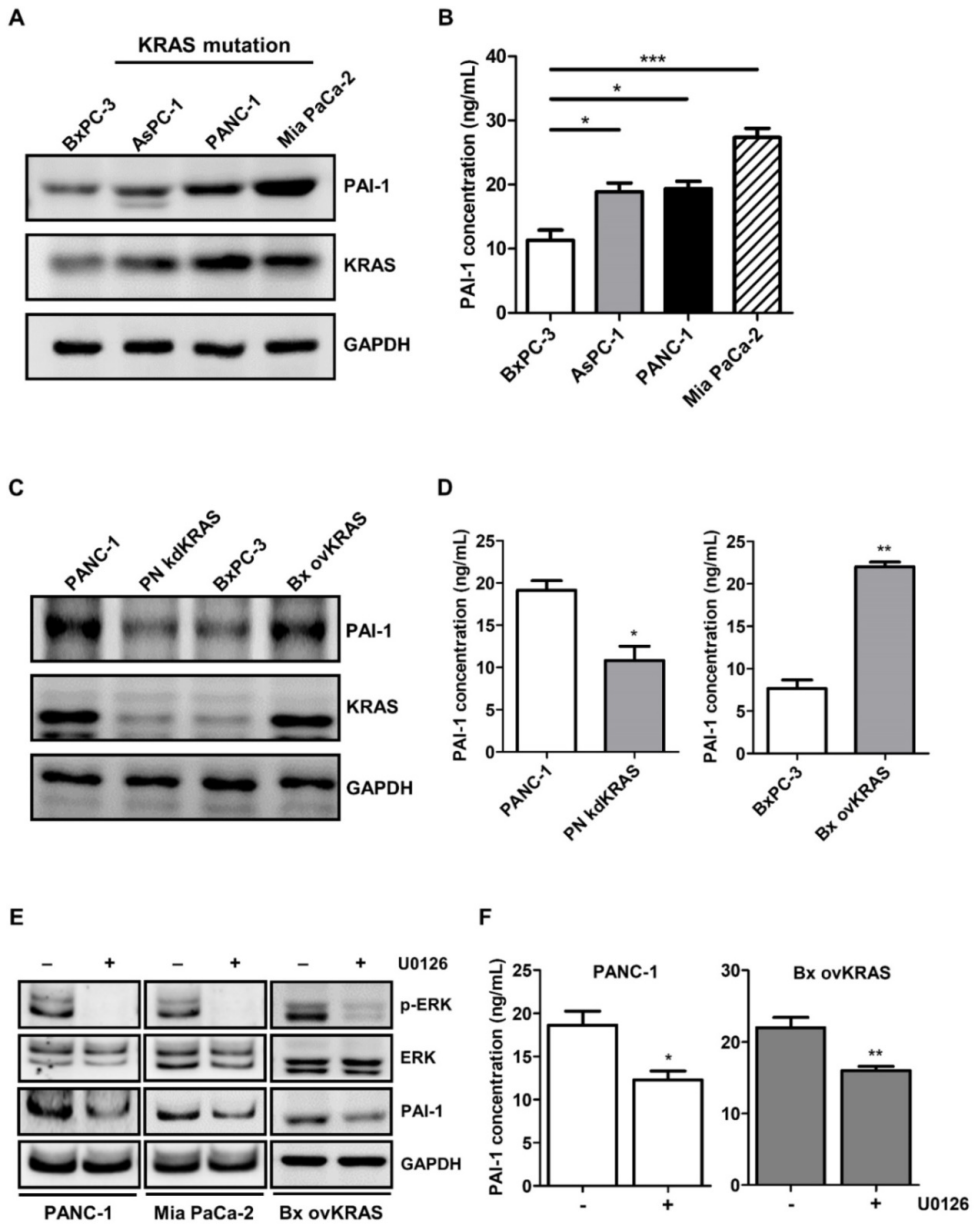
** PAI-1 expression in pancreatic cancer cells is upregulated by the KRAS/ERK pathway. A,** PAI-1 protein expression in different pancreatic cancer cell lines was determined by Western blotting. **B,** PAI-1 concentration in the CM of different pancreatic cancer cell lines was measured by ELISA. The bar graph depicts PAI-1 concentration in culture medium. * *P* < 0.05; *** *P* < 0.001, significant difference between cell lines, one-way ANOVA. **C,** PAI-1 and KRAS expression in PANC-1, KRAS knockdown PANC-1 (PN kdKRAS), BxPC-3, and BxPC-3 overexpressing mutant KRAS (Bx ovKRAS) was compared using Western blotting. **D,** PAI-1 secretion in the CM of PANC-1, PN kdKRAS, BxPC-3, and Bx ovKRAS was analyzed by ELISA. The bar graphs depict PAI-1 concentration in the CM. * P < 0.05; ** P < 0.01 versus parental control cells, unpaired t-test. **E and F,** After treatment with the MEK/ERK inhibitor U0126 (20 μg/mL) for 24 hours, expression of ERK, phosphorylated ERK, and PAI-1 in PANC-1, Mia PaCa-2, and Bx ovKRAS cells was analyzed by Western blotting, and PAI-1 levels in the CM of PANC-1 and Bx ovKRAS cells were determined by ELISA. The bar graphs depict PAI-1 concentration in the CM. * P < 0.05 versus unstimulated controls, unpaired t-test.

PAI-1 expression is reportedly regulated by the RAS/MAPK pathway 31. Given that activating *KRAS* mutations are the most prevalent oncogenic driver in pancreatic cancer 32, we investigated the role of KRAS in the regulation of PAI-1 expression. PAI-1 expression was screened in several human pancreatic cancer cell lines. Higher expression of PAI-1 was seen in AsPC-1, PANC-1, and Mia PaCa-2 cells that harbor *KRAS* mutations than in wild-type KRAS expressing BxPC-3 cells (Figure 4A and 4B), suggesting a positive correlation between KRAS activity and PAI-1 expression. To verify whether PAI-1 expression is regulated by KRAS, stable KRAS knockdown PANC-1 cells (PN kdKRAS cells) and wild-type KRAS overexpressing BxPC-3 cells (Bx ovKRAS cells) were established to determine their PAI-1 expression. The expression of PAI-1 was decreased in PN kdKRAS cells as compared with control PANC-1 cells. By contrast, PAI-1 was upregulated in Bx ovKRAS cells as compared with their corresponding control (Figure 4C and 4D). Because ERK is the most common effector downstream of KRAS, we determined whether KRAS upregulates PAI-1 via ERK in pancreatic cancer cells. In PANC-1, Mia PaCa-2, or Bx ovKRAS cells, suppression of ERK phosphorylation by the MEK/ERK inhibitor U0126 reduced PAI-1 expression (Figure 4E). The concentrations of PAI-1 in the culture media of Bx ovKRAS and PANC-1 cells were also decreased after U0126 treatment (Figure 4F). Taken together, PAI-1 is a potential downstream target of the KRAS/ERK pathway in pancreatic cancer cells.

### PAI-1 stimulates the activation of PSCs by binding to LRP-1

**Figure 5:**
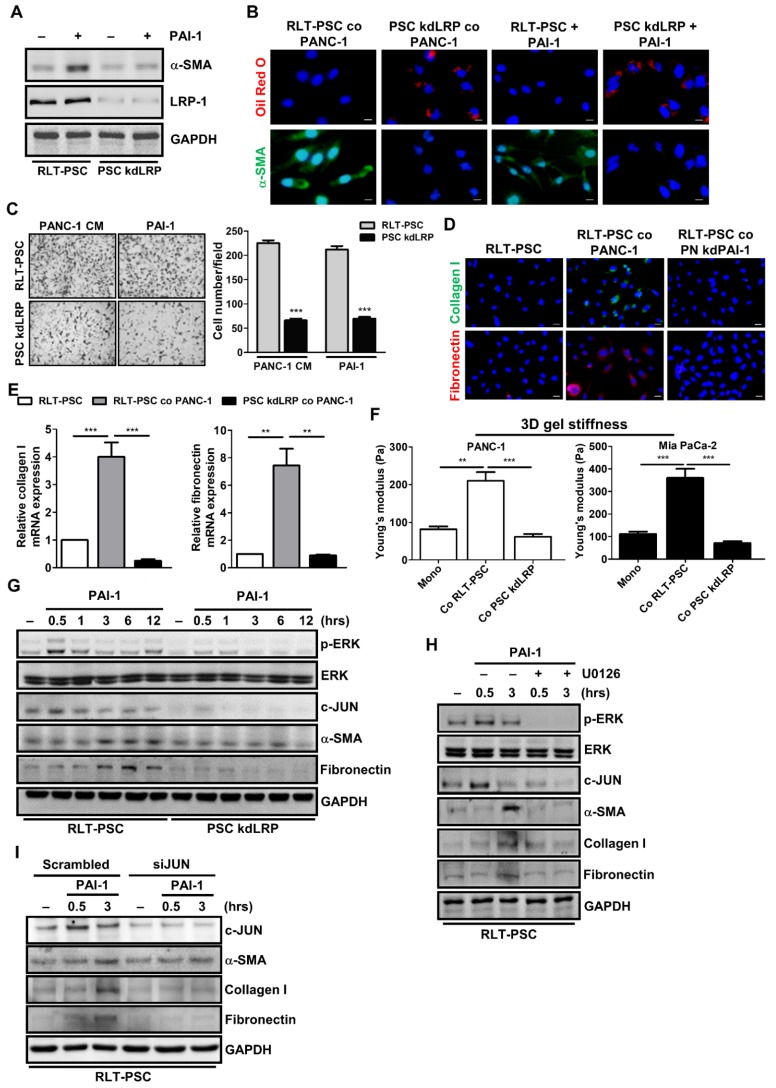
** LRP-1/ERK/c-JUN signaling is required for PAI-1-dependent PSC activation. A,** After PAI-1 (200 ng/mL) treatment, α-SMA expression in RLT-PSC and PSC kdLRP cells was determined by Western blotting. **B,** After coculture with PANC-1 cells for 72 hours or treatment with PAI-1 (200 ng/mL) for 24 hours, RLT-PSC and PSC kdLRP cells were stained with anti-α-SMA (green) and oil red O (red). Magnification: 400x, scale bar: 20 μm. **C,** The migration of RLT-PSC cells and PSC kdLRP cells was analyzed using transwell assays after incubation with CM derived from PANC-1 or fresh medium containing PAI-1 (200 ng/mL) for 24 hours. The bar graph indicates the average number of migrating cells. *** P < 0.001 versus scrambled control cells, unpaired t-test. **D and E,** Protein and mRNA expression of collagen I and fibronectin in RLT-PSC cells and PSC kdLRP cells was determined by IF staining and q-PCR, respectively. The bar graph depicts relative mRNA expression of collagen I and fibronectin. ** *P* < 0.01; *** *P* < 0.001, significant difference between groups, one-way ANOVA. **F,** After coculture of cancer cells with RLT-PSC cells or PSC kdLRP cells, the stiffness of 3D coculture gels was measured by AFM. The bar graphs indicate the average values of Young's modulus of organotypic gels.** *P* < 0.01; *** *P* < 0.001, significant difference between groups, one-way ANOVA. **G,** After treatment with PAI-1 (200 ng/mL) for the indicated times in RLT-PSC cells and PSC kdLRP cells, expression of the indicated proteins was determined by Western blot analysis. **H,** RLT-PSC cells and PSC kdLRP cells were pretreated with U0126 (20 μg/mL) for 1 hour followed PAI-1 stimulation for 0.5 and 3 hours. Cell lysates were subjected to Western blotting with antibodies against the indicated proteins. **I,** RLT-PSC cells were transiently transfected with either nontargeting control siRNA or *JUN* siRNA. Forty-eight hours after transfection, cells were treated with PAI-1 (200 ng/mL) for 0.5 and 3 hours, and then cell lysates were subjected to Western blotting using antibodies against the indicated proteins.

LRP-1 belongs to the low-density lipoprotein receptor family that mediates the internalization of various extracellular macromolecules. In addition to mediating endocytosis, LRP-1 also functions as a signaling receptor in multiple cellular activities 33. Interestingly, the promigratory function of PAI-1 requires LRP-1-dependent signaling 34. To investigate whether LRP-1 plays a role in PAI-1-mediated PSC activation, we established stable LRP-1 knockdown RLT-PSCs (PSC kdLRP). After PAI-1 treatment, expression of α-SMA was increased in control RLT-PSCs, but was not changed in PSC kdLRP cells (Figure 5A). IF staining also shows that coculture with PANC-1 cells or treatment with PAI-1 increased α-SMA expression and decreased the level of vitamin A-containing lipid droplets in parental RLT-PSCs but not in PSC kdLRP cells (Figure 5B). Furthermore, treatment with PANC-1 CM or PAI-1 induced migration of RLT-PSCs, but this effect was blocked by knockdown of LRP-1 (Figure 5C). Knockdown of LRP-1 in RLT-PSCs also counteracted coculture-mediated increase in expression of collagen I and fibronectin (Figure 5D and 5E) and gel stiffness (Figure 5F).

### PAI-1 induces PSC activation through ERK/c-JUN signaling

Because ERK serves as a key downstream mediator of LRP-1-regulated invasion and migration in cancer cells 35, we investigated whether PAI-1 induces PSC activation by triggering LRP-1/ERK signaling. After PAI-1 treatment, ERK was phosphorylated with a peak level at the time point of 30 minutes, and α-SMA as well as fibronectin were upregulated after 3 hours in RLT-PSCs but not in PSC kdLRP cells (Figure 5G). Suppression of ERK phosphorylation by U0126 in RLT-PSCs decreased expression of α-SMA, collagen I, and fibronectin (Figure 5H). We further identified that c-JUN, a downstream effector of ERK, is involved in PAI-1-induced PSC activation because c-JUN expression was upregulated by PAI-1 in RLT-PSCs (Figure 5G). Treatment with U0126 abolished PAI-1-mediated upregulation of c-JUN (Figure 5H). Knockdown of c-JUN in RLT-PSCs by siRNA inhibited PAI-1-induced expressions of α-SMA, collagen I, and fibronectin (Figure 5I). Taken together, these results suggest that PAI-1 can activate the LRP-1/ERK/c-JUN pathway to induce PSC activation.

### Activated PSCs potentiate the aggressiveness of pancreatic cancer via IL-8

**Figure 6:**
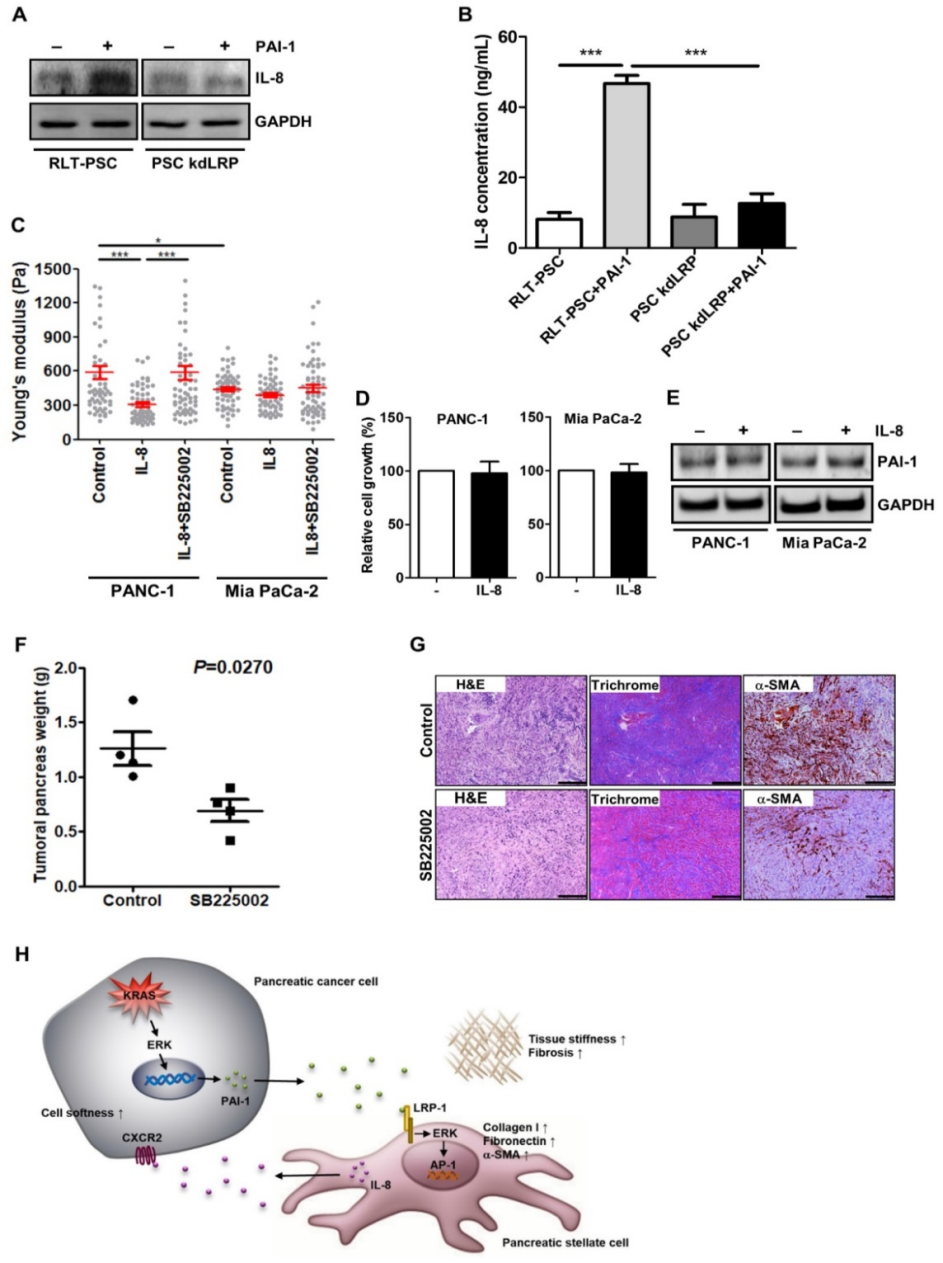
** Activated PSCs enhance pancreatic cancer aggressiveness through paracrine IL-8**.** A,** After PAI-1 treatment, cell lysates of RLT-PSC and PSC kdLRP were prepared and subjected to Western blotting for measurement of IL-8 expression. **B,** RLT-PSC and PSC kdLRP cells were treated with PAI-1. The CM of these cells were collected to measure IL-8 levels using ELISA. The bar graph depicts IL-8 concentration in the CM. *** *P* < 0.001, significant differences between groups, one-way ANOVA. **C,** PANC-1 and Mia PaCa-2 cells were treated with IL-8 (10 ng/mL) or IL-8 in combination with the CXCR2 antagonist SB225002 (200 nM). The stiffness of cancer cells was measured with AFM. The Young's modulus of each individual cell was plotted. * *P* < 0.05; *** *P* < 0.001, significant difference between groups, two-way ANOVA. **D,** Forty-eight hours after IL-8 treatment, the growth of PANC-1 and Mia PaCa-2 cells was analyzed by MTT assay.** E,** After IL-8 treatment, PAI-1 expression in PANC-1 and Mia PaCa-2 cells was determined by Western blotting. **F,** Four weeks old KPC mice were treated or not for 4 weeks with SB225002. At the end of experiment, tumors were removed and their weights were measured and compared. The dots on the graph indicate each individual tumor weight. P=0.027, unpaired t-test. **G,** The levels of fibrosis and α-SMA in KPC tumor tissues were determined by Masson's trichrome stain and IHC, respectively. Magnification: 40x, scare bar: 500 μm. **H,** A schematic model illustrating the molecular mechanism by which PAI-1 regulates the tumor-stroma interactions and TME remodeling.

Proinflammatory cytokine IL-8 with protumoral activities is frequently expressed in pancreatic tumor stroma 36, where activated PSCs are the major producer of cytokines, including IL-8 37. Together with our cytokine array data that IL-8 level was increased in coculture medium (Figure 2E), we assumed that activated PSCs may release IL-8 to affect cancer cell behavior. In RLT-PSCs, PAI-1 treatment indeed induced IL-8 expression and secretion, but the induction was inhibited by knockdown of LRP-1 (Figure 6A and 6B), demonstrating the upregulation of IL-8 in activated PSCs. To determine whether PSCs promoted pancreatic cancer cell invasion into the 3D gel (Figure 2D) through IL-8, we next examined the effect of IL-8 on the softness of cancer cells, which has been associated with a more invasive phenotype and metastatic potential of tumor cells 38, 39. PANC-1 cells became softer after IL-8 treatment (Figure 6C). By contrast, in Mia PaCa-2 cells that are highly malignant, IL-8 treatment could induce only a slight increase in cell softness (Figure 6C). Inhibition of IL-8 receptor CXCR2 in both cell lines with the small molecule inhibitor SB225002 attenuated IL-8-mediated increase in cell softness (Figure 6C). We examined whether IL-8 also has effects on cell growth and PAI-1 expression in pancreatic cancer cells to promote fibrosis, but we observed that cell growth and PAI-1 expression did not change after IL-8 treatment (Figure 6D and 6E). Furthermore, we assessed the *in vivo* antitumor effects of blocking IL-8 signaling and found that the mean tumor weight of KPC mice receiving SB225002 was lower than that of controls (Figure 6F). Interestingly, Masson's trichrome staining and IHC staining show that treatment with SB225002 also reduced fibrosis and α-SMA expression in KPC tumor tissues (Figure 6G), demonstrating a reciprocal interaction between pancreatic cancer cells and PSCs.

## Discussion

Severe fibrosis in pancreatic cancer can retard chemotherapy drug penetration and uptake into tumor tissues and has been considered one of the causes for failure of clinical treatment in pancreatic cancer. PSCs not only represent the key effector cells in pancreatic fibrosis but also interact with cancer cells to create a permissive microenvironment for cancer progression, which provides a strong rationale for depleting PSCs as a promising therapeutic approach to improve pancreatic cancer treatment. Here, we reported that high levels of activated PSCs in pancreatic tumor tissues were associated with increased tumor stiffness and poor patient outcomes. PSC activation after coculture with pancreatic cancer cells resulted in increased ECM protein production and increased organotypic coculture gel stiffness. The activation of PSCs was mediated by pancreatic cancer cells-derived PAI-1 acting through the LRP-1/ERK/c-JUN pathway. Activated PSCs secreted high levels of IL-8 that had protumor effects on pancreatic cancer cells. Our study highlights potential molecules involving in the interactions between pancreatic cancer cells and PSCs.

It is well known that PAI-1 can neutralize activities of tPA and uPA to impede plasmin formation and protect ECM proteins from proteolytic degradation 17. Therefore, a sustained increase in PAI-1 level may result in collagen accumulation and thus tissue fibrosis 40. Recently, PAI-1 has also been linked to cancer. PAI-1 deficiency in transgenic mice reduced tumor growth and angiogenesis 41. Elevated PAI-1 expression in many cancers, such as glioma, ovarian cancer, and pancreatic cancer, was associated with unfavorable patient outcomes 42, 43, suggesting its potential role in tumor promotion. In this study, we have found that pancreatic cancer cells-secreted PAI-1 could stimulate PSC activation to produce ECM proteins, which might consequently result in stromal fibrosis and tumor progression. To the best of our knowledge, this is the first study to uncover a previously unrecognized function of PAI-1 as a PSC activator and suggest that interfering with PAI-1 signaling may be an effective antifibrotic treatment.

Over the last few decades, intensive attention has been focused on the interaction between the TME and cancer cells. Research in the field of mechanobiology shows that cellular activities required for cancer formation, such as growth, differentiation, motility, and invasion, can be affected by physical interactions between cells and their ECM adhesions 44. For example, collagen crosslinking could stiffen ECM and force breast malignancy 45. Increasing ECM rigidity strongly enhanced glioma cell proliferation and motility 46. Consistently, in our study, PSC activation increased the aggressiveness of pancreatic cancer cells and tumor stiffness, contributing to dismal survival of pancreatic cancer patients. However, unexpected roles for myofibroblasts in pancreatic cancer have recently been highlighted by two groups 47, 48. They found that the reduction of myofibroblasts and fibrosis in pancreatic cancer did not improve the treatment efficacy of gemcitabine and even accelerated tumor growth. The controversy may arise from the heterogeneity of PSCs that consist of distinct subpopulations having different functions in pancreatic cancer. Recent research has shown that CD10^+^ PSCs found in great abundance in pancreatic cancer significantly increased cancer cells growth and invasiveness 49. Another population of CD271^+^ PSCs were frequently found in normal pancreatic tissues, and high CD271 expression in pancreatic tumors was associated with good prognosis 50. The subpopulations of PSCs that contribute to fibrosis and cancer progression need to be identified in the future.

In addition to the depletion of stromal cells, ECM proteolytic degradation by enzymes is regarded as a promising way to reduce fibrosis and enhance drug perfusion. The polysaccharide hyaluronic acid (HA), a major component of ECM, frequently accumulates in malignant tumors, which creates a favorable microenvironment for cancer progression 51. In pancreatic cancer, the accumulation of HA favors epithelial-mesenchymal transition and tumor growth, suggesting that HA represents a potential therapeutic target 52. Several stroma-targeted agents have so far been developed at the preclinical or clinical stage, of which PEGPH20 is the best characterized. Administration of PEGPH20 ablated stromal HA, normalized interstitial fluid pressure, allowed reexpansion of collapsed tumor microvasculature, and consequently enhanced the tumoricidal activity of chemotherapy in preclinical models 53, 54. Because depletion of myofibroblasts in pancreatic cancer reduced fibrosis but not affected HA content 47, it is reasonable to infer that a triple combination of PSC-targeting agents, HA-targeting enzymatic agents, and chemotherapeutic drugs may be an effective therapeutic option in pancreatic cancer.

In conclusion, we have identified PAI-1 as a novel regulator in the interaction between pancreatic cancer cells and PSCs (Figure 6H). Pancreatic cancer cells-derived PAI-1 can induce PSC activation to promote fibrosis and tumor stiffness and release IL-8 to enhance the malignant phenotype of cancer cells. Accordingly, blockade of PAI-1 signaling is a theoretically promising approach to resolve fibrosis and improve pancreatic cancer treatment.

## Supplementary Material

Supplementary figures and tables.

## Abbreviations

PSCs: pancreatic stellate cells
ECM: extracellular matrix
α-SMA: α-smooth muscle actin
TME: tumor microenvironment
PAI-1: plasminogen activator inhibitor-1
SERPIN: serine protease inhibitor
LRP-1: low-density lipoprotein receptor-related protein-1
tPAR: tissue-type plasminogen activator receptor
uPAR: urokinase-type plasminogen activator receptor
CM: conditioned medium
FBS: fetal bovine serum
NAC: N-acetylcysteine
TMA: tissue microarray
IP: intraperitoneal
NCKUH: National Cheng Kung University Hospital
IHC: immunohistochemistry
MTT: 3-(4,5-dimethylthiazol-2-yl)-2,5-diphenyl-tetrazolium bromide
IF: immunofluorescence
AFM: atomic force microscopy
OS: overall survival
DFS: disease-free survival
HA: hyaluronic acid
