# Supplementary material for: Pancreatic stellate cells activated by mutant KRAS-mediated PAI-1 upregulation foster pancreatic cancer progression via IL-8
Source: Theranostics. 2019 Sep 23;9(24):7168–83. doi: 10.7150/thno.36830 (PMC6831292; doi:10.7150/thno.36830)
Supplement: Supplementary file 1 — Supplementary figures and tables. [file thnov09p7168s1.pdf]

Supplementary Table S1. Clinical parameters of pancreatic cancer patients in TMAs

Variable	No. of Patients (%)
**Sex**	
Men	60 (65.9)
Women	31 (34.1)
**Age**	
≦65	46 (50.5)
>65	45 (49.5)
**Tumor location**	
Head	55 (60.4)
Neck	7 (7.7)
Body/tail	16 (17.6)
Uncinate process	13 (14.3)
**Tumor size, cm**	
≦3	48 (52.7)
>3	43 (47.3)
**Margin status**	
R0	64 (70.3)
R1	23 (25.3)
R2	4 (4.4)
**Tumor grade**	
Poorly differentiated	17 (18.7)
Moderately differentiated	50 (54.9)
Well differentiated	24 (26.4)
**Stage**	
I	11 (12.1)
II	74 (81.3)
III	4 (4.4)
IV	2 (2.2)
**Recurrence status**	
Yes	61 (67.0)
No	30 (33.0)
**Metastasis status**	
Yes	68 (74.7)
No	23 (25.3)
**Lymph node status**	
Negative	44 (48.4)
Positive	47 (51.6)
**Adjuvant therapy**	
Yes	37 (40.7)
No	54 (59.3)
**CA19-9, U/mL**	
≦37	19 (20.9)
>37	72 (79.1)
